# Factors associated with anaemia among preschool- age children in underprivileged neighbourhoods in Antananarivo, Madagascar

**DOI:** 10.1186/s12889-022-13716-6

**Published:** 2022-07-09

**Authors:** Mirella Malala Randrianarisoa, Maheninasy Rakotondrainipiana, Ravaka Randriamparany, Prisca Vega Andriantsalama, Anjasoa Randrianarijaona, Azimdine Habib, Annick Robinson, Lisette Raharimalala, Francis Allen Hunald, Aurélie Etienne, Jean-Marc Collard, Frédérique Randrianirina, Robert Barouki, Clement Pontoizeau, Alison Nestoret, Nathalie Kapel, Philippe Sansonetti, Pascale Vonaesch, Rindra Vatosoa Randremanana

**Affiliations:** 1 Institut Pasteur de Madagascar, Unité Epidémiologie et de Recherche Clinique, BP 1274, Ambatofotsikely, 101 Antananarivo, Madagascar; 2 Centre Hospitalier Universitaire Mère Enfant de Tsaralalana, rue Patrice Lumumba, Rue Mabizo S, 101 Antananarivo, Madagascar; 3 Centre de Santé Maternelle et Infantile de Tsaralalana, Lalana Andriantsilavo, 101 Antananarivo, Madagascar; 4 Service de Chirurgie pédiatrique, Centre Hospitalier Universitaire Joseph Ravoahangy Andrianavalona, BP 4150, Ampefiloha, 101 Antananarivo, Madagascar; 5 The Center for Microbes, Development and Health, Institut Pasteur of Shanghai/Chinese Academy of Sciences, Shanghai, China; 6 Laboratoire de Biochimie Métabolomique et Protéomique, Hôpital Universitaire Necker-Enfants Malades, Paris, France; 7 Service de Coprologie Fonctionnelle, Hôpital Salpétrière Paris, Paris, France; 8 Unité de Pathogénie Microbienne, Institut Pasteur, 25-28 Rue du Dr Roux, Paris, France; 9 Department of Fundamental Microbiology, University of Lausanne, Campus UNIL-Sorge, 1015 Lausanne, Switzerland

**Keywords:** Anaemia, Factors, Underprivileged neighbourhoods, Children, Antananarivo

## Abstract

**Background:**

Anaemia occurs in children when the haemoglobin level in the blood is less than the normal (11 g/dL), the consequence is the decrease of oxygen quantity in the tissues. It is a prevalent public health problem in many low-income countries, including Madagascar, and data on risk factors are lacking. We used existing data collected within the pathophysiology of environmental enteric dysfunction (EED) in Madagascar and the Central African Republic project (AFRIBIOTA project) conducted in underprivileged neighbourhoods of Antananarivo to investigate the factors associated with anaemia in children 24 to 59 months of age.

**Methods:**

Children included in the AFRIBIOTA project in Antananarivo for whom data on haemoglobin and ferritin concentrations were available were included in the study. Logistic regression modelling was performed to identify factors associated with anaemia.

**Results:**

Of the 414 children included in this data analysis, 24.4% were found to suffer from anaemia. We found that older children (adjusted OR: 0.95; 95% CI: 0.93–0.98) were less likely to have anaemia. Those with iron deficiency (adjusted OR: 6.1; 95% CI: 3.4–11.1) and those with a high level of faecal calprotectin (adjusted OR: 2.5; 95% CI: 1.4–4.4) were more likely to have anaemia than controls.

**Conclusions:**

To reduce anaemia in the children in this underprivileged area, more emphasis should be given to national strategies that improve children’s dietary quality and micronutrient intake. Furthermore, existing measures should be broadened to include measures to reduce infectious disease burden.

**Supplementary Information:**

The online version contains supplementary material available at 10.1186/s12889-022-13716-6.

## Introduction

Anaemia is a prevalent public health problem in low-income countries. Anaemia has diverse consequences for human health and development. It has been associated with low birth weight, premature birth, and increased child morbidity and mortality as well as with delayed cognitive development, poor physical growth, poor work productivity and low income in adulthood [1–5].

In children < 5 years of age, anaemia is defined as a blood haemoglobin concentration lower than 110 g/l. It affects approximately 43% of preschool-aged (PreSAC) children worldwide [6, 7]. Among this population group, anaemia is a severe public health problem; the World Health Organization (WHO) reports a prevalence of ≥40% in almost all WHO member states in the African region [6]. A recent meta-analysis of data on African children reported that the risk of infant mortality decreases by 24% with an increase of 10 g/l in haemoglobin (Hb) concentration [8]. The youngest age group (< 5 years) had the least favourable changes in anaemia prevalence between 1990 and 2010; indeed, it was the only age group with an increased anaemia prevalence during this period [9]. In low-income and middle-income countries (LMICs), the immediate causes of anaemia can be grouped into three categories: nutritional deficiencies (iron, vitamins A and B12, riboflavin, folate and other micronutrient deficiencies), inflammation and infections (e.g., soil-transmitted helminth infections, malaria, tuberculosis), and genetic haemoglobin (Hb) disorders (sickle cell disease, thalassaemias, and other disorders) [4]. Worldwide, it is estimated that the top two specific causes of anaemia in both sexes and all ages from 1990 to 2019 were dietary iron deficiency, as well as hemoglobinopathies and hemolytic anaemias [10].

Anaemia also has many interrelated distal determinants such as food insecurity, inadequate access to water and sanitation, inadequate maternal and child care, inadequate knowledge of health/nutrition, inadequate education and limited access to health/nutrition services [4, 11, 12].

In Madagascar, a very low-income country with a gross national annual income per capita of 400 USD, the prevalence of nutritional problems such as anaemia is high. In a recent survey, half of the PreSAC (50.3%) were anaemic [9, 10], a situation that calls for urgent responses by the government [13]. There is a lack of study which assess the prevalence of anaemia and associated factors in Madagascar. The availability of local information on prevalence and related risk factors could help decision-makers to improve or strengthen interventions for the control of anaemia. We used data collected in underprivileged areas of Antananarivo during the AFRIBIOTA study to assess factors associated with the occurrence of anaemia. The AFRIBIOTA study is a case–control study that uses a variety of approaches and disciplines to understand the personal and environmental context that leads to and maintains EED and growth delay [14]. AFRIBIOTA was conducted in Bangui, the capital of the Central African Republic (CAR), and Antananarivo, the capital of Madagascar. This data analysis will be important in designing and targeting approaches to improve the nutritional status of children in these underprivileged areas.

## Methods

### Data source

This study conducts a secondary analysis of data collected as part of the AFRIBIOTA project, a translational study of the pathophysiology of EED performed in the two African cities of Antananarivo (Madagascar) and Bangui (Central African Republic). Details of the project objectives and methodology of the AFRIBIOTA project are provided elsewhere [14]. AFRIBIOTA is a case–control study of stunting in which 260 stunted children and 200 age- and sex-matched nonstunted children were recruited in each country. Data collection for the AFRIBIOTA project was conducted from November 2016 to March 2018. Children from 24 to 59 months of age with no obvious signs of severe disease and with negative HIV serology were recruited. The recruitment was mainly community-based and was conducted in underprivileged areas of the Urban Commune of Antananarivo (Andranomanalina Isotry, Ankasina and their surrounding neighbourhoods) and in three health care facilities (The Centre de Santé Maternelle et Infantile de Tsaralalana (CSMI), the Centre Hospitalier Universitaire Mère Enfant de Tsaralalana and the paediatric surgery department of the Centre Hospitalier Universitaire Joseph Ravoahangy Andrianavalona).

### Study design/recruitment

**Fig. 1:**
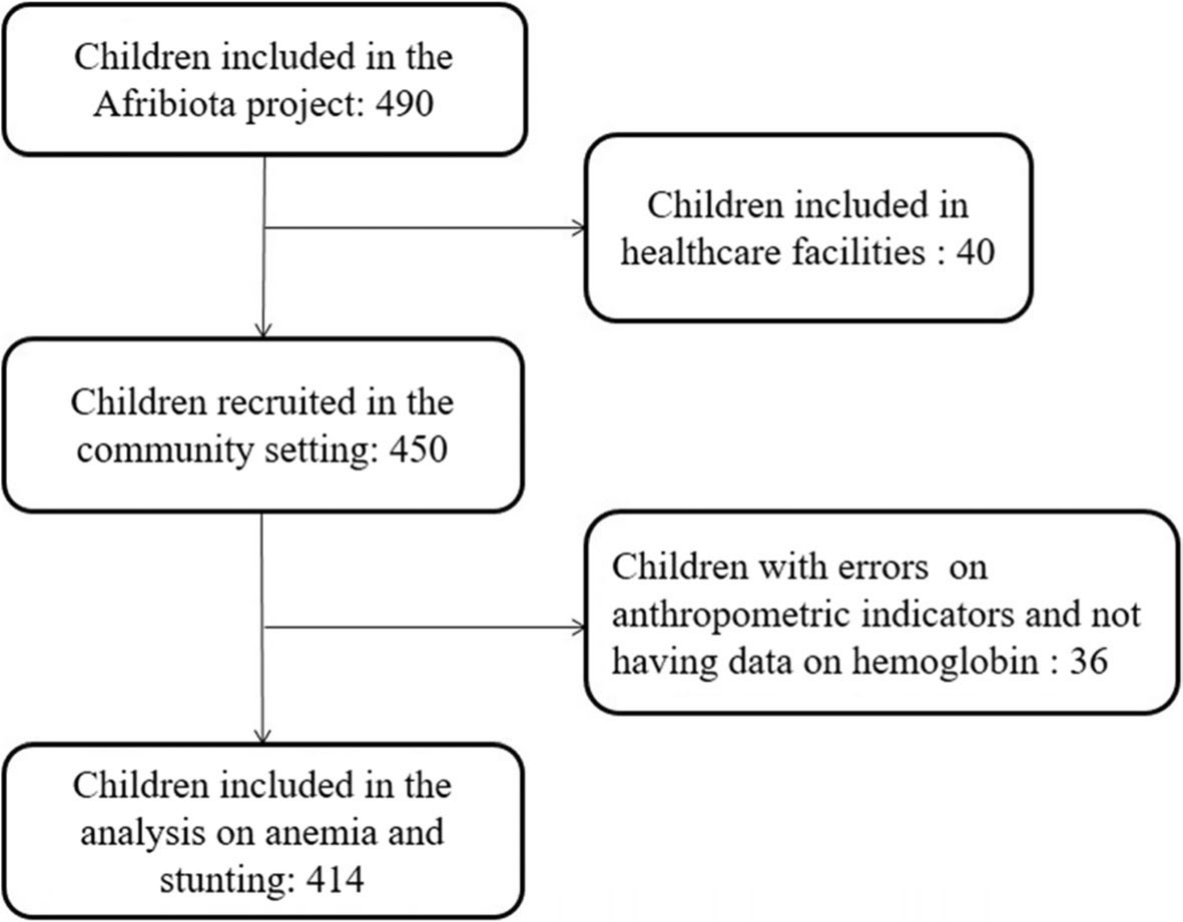
Flow chart of the study participants

This secondary data analysis focuses on the children living in Antananarivo who were recruited from the community setting. Children included in the AFRIBIOTA project in Antananarivo and for whom data on haemoglobin and ferritin concentrations were available were included in this secondary analysis (Fig. 1).

### Data collection

Data were collected by interviewing mothers/closest caregivers and using a standardized questionnaire. Anthropometric measurements were performed by trained health professionals; blood and stool samples were also collected. Screening and recruitment were conducted at the community level with the support of community health workers. The interviews and the collection of biological samples were conducted at the hospital centres: Centre Hospitalo-Universitaire Mère Enfant de Tsaralalana and Centre Hospitalo-Universitaire Joseph Ravoahangy Andrianavalona.

#### Anthropometric measurements

Each child’s weight was measured twice to the nearest 0.1 kg using an electronic scale (KERN, ref. MGB 150 K100 and EKS, People’s Republic of China). When the difference in the two measurements exceeded 0.1 kg, another measurement was performed until the last three values did not differ by more than 0.1 kg. Each child’s height was measured to the nearest 0.1 cm with the child in a standing position using collapsible height boards (ShorrBoard® Infant/Child/Adult Measuring Board, MD, USA). The same procedure was followed for each child to ensure consistent measurement. For both indicators, the mean of the two or three values obtained was reported.

#### Blood sample collection

Venous blood samples (2 mL) were collected and used in complete blood count, C-reactive protein (CRP), ferritin and citrulline analysis. They were collected in Microtainer**®** tubes containing ethylenediamine tetraacetic acid (EDTA) and sent at + 4 °C to the Clinical Biology Center of the Institut Pasteur de Madagascar (IPM) within 1 hour after blood collection. One hundred microlitres (100 μL) of plasma was extracted from each sample of whole blood, stored at − 80 °C and sent to the Hôpital Universitaire Necker-Enfants Malades, Paris for citrulline testing.

#### Stool sample collection

A clean, dry plastic container was given to the mother/caregiver of each child for stool sample collection with detailed instructions on how to collect fresh stool samples. Part of each stool sample was sent to the Unité de Bactériologie expérimentale at IPM as soon as possible for the detection of intestinal parasites. The remainder of each stool sample was stored in liquid nitrogen in the field and shipped to IPM for storage at − 80 °C. An aliquot of each sample was shipped on dry ice to the Service de Coprologie Fonctionnelle, Hôpital Salpétrière Paris for measurement of calprotectin and alpha-antitrypsin levels.

#### Questionnaire

The questionnaire collected individual data about each child (diseases requiring hospital admission during the year prior to the survey, feeding practices (age at introduction of complementary feeding, age at cessation of breastfeeding, 24-hour recall)) and about the child’s mother (education level, nutritional status). Household data, including type of housing and amount of household assets, were also collected. A detailed description of the questionnaire is given in [15].

#### Laboratory analyses

The complete blood count, including haemoglobin assessment, was performed on a SYSMEX autoanalyser (XN 1000 or XT-2000 i) (Landskrona, Sweden) using the fluorocytometric technique. Plasma CRP concentrations were assessed using an enzyme-linked immunosorbent assay (ELISA). Plasma ferritin concentrations were assessed on the ARCHITECT machine (Abbott, IL, USA) using a chemiluminescent microparticle immunoassay (CMIA). These analyses were performed according to standard procedures at the Clinical Biology Centre of IPM (ISO18189 certification).

Citrulline was measured by liquid chromatography coupled to tandem mass spectrometry (UPLC–MS/MS) at the Laboratoire de Biochimie Métabolomique et Protéomique, Hôpital Universitaire Necker-Enfants Malades, Paris. For accurate quantification, a stable isotope internal standard of the same structure (purchased from Eurisotop, Saint Aubin, France) was added to the sample before protein precipitation. Before analysis, the samples were derivatized using the AccQ Tag™ Ultra (Waters Corporation, Milford, MA, USA) according to the manufacturer’s recommendations. Amino acid separation was performed on an Acquity™ UPLC system using a CORTECS™ UPLC C18 column (1.6 μm, 2.1 × 150 mm) coupled to a microTQS™ tandem mass spectrometer (Waters Corporation, Milford, MA, USA). Faecal calprotectin was assayed using a “sandwich”-type ELISA that uses a polyclonal Ab system (Calprest; Eurospital). The concentration of α1 antitrypsin (AAT) in faeces was measured using an immunonephelemetric method adapted on the BN ProSpec system (Siemens) [16]. The analysis of these faecal biomarkers was conducted at the Service de Coprologie Fonctionnelle, Hôpital Salpétrière Paris.

All faecal samples were physically examined and screened for intestinal parasites as previously described [17].

#### Definition of outcome and covariates

The main variable of interest was the occurrence of anaemia. Anaemia was defined according to the WHO criteria [18] as Hb less than 110 g/l (adjusted for altitude). Age, sex and height and the 2006 WHO Child Growth Standards for children 24 to 59 months of age [19] were used to calculate children’s height-for-age z scores, which were used to define stunting and normal growth. Stunting and normal growth were defined as height-for-age z score < − 2 SD and height-for-age z score > − 2 SD, respectively. Anaemia was defined as severe when the child’s Hb level was less than 70 g/l and moderate at Hb levels between 70 g/l and 99 g/l. Anaemia was defined as mild if the child’s Hb level was between 100 g/l and 109 g/l [1].

A dietary diversity score (DDS) was calculated by counting the number of food groups consumed by the child during the 24-hour period prior to the survey. The WHO recommends basing the DDS on seven food groups: (1) grains, roots and tubers; (2) legumes and nuts; (3) dairy products; (4) flesh foods (meats/fish/poultry); (5) eggs; (6) vitamin A-rich fruits and vegetables; and (7) other fruits and vegetables. A diverse diet is defined as one that has a DDS of at least four. Accordingly, children with a DDS *<* 4 were classified as having low dietary diversity; otherwise, they were considered to have an adequate diet [20].

The body mass index (BMI) of the mothers was assessed by dividing their weight (in kilograms) by the square of their body height (in metres). Mothers were classified as underweight if their BMI was < 18.5 kg/m^2^ and as not underweight if their BMI was ≥18.5 kg/m^2^. Pregnant mothers were classified according to the categories proposed by Ververs et al. [21]. A wealth index based on a minimal set of assets was created, allowing separation of the subjects into three distinct groups based on principal component analysis (PCA). The minimal set of assets included housing materials (floor and wall materials, ownership of an automobile, telephone, bicycle, motorcycle), access to specific utilities (electricity, plumbing, cooking location), and family size. We defined three household wealth categories according to the clusters observed: the poorest, middle and wealthiest categories. Details of the wealth index have been described previously [15].

Iron deficiency was defined as a plasma ferritin concentration < 12 μg/l in the absence of inflammation [22]. To eliminate the influence of inflammation on ferritin plasma concentrations, a correction factor of 0.67 was used to adjust the ferritin plasma concentration value in the presence of inflammation [23]. A CRP value > 6 mg/l was considered an indicator of inflammation. For citrulline, a value below 7 μmol/l was considered too low, and a value above 43 μmol/l was considered too high according to the normal values provided by the Hôpital Necker Enfants Malades. According to the thresholds used in routine diagnostics at the Hôpital Pitié-Salpêtrière, the threshold for AAT was 1.25 mg/g dry weight, and values above this threshold were considered elevated. For calprotectin, the normal value was equal to or less than 150 μg/g for children 2–3 years of age and equal to or less than 100 μg/g for those between 3 and 5 years of age; children who had values above these thresholds were classified as having elevated values.

#### Statistical analysis

Statistical analysis was performed using R statistical software (version 3.4.3; The R Foundation for Statistical Computing, Vienna, Austria). Descriptive analysis was performed using proportions for categorical variables and means or medians with interquartile ranges for continuous variables according to their distributions.

We used binomial logistic regression model analysis to identify independent predictors of the occurrence of anaemia. A bivariate analysis was performed to identify the explanatory variables to be included in the multivariate analysis. All explanatory variables with *p* value < 0.20 in the bivariate analysis were included in the logistic regression model. A backwards stepwise logistic regression was applied to obtain the variables associated with the occurrence of anaemia. Explanatory variables included the following: 1) biological characteristics: iron status, presence of intestinal parasites, alpha-antitrypsin and calprotectin levels, status of intestinal damage and repair (citrulline levels in blood); 2) child characteristics: age, gender, nutritional status, occurrence of dental caries or symptoms such as dermatitis, cough, runny nose, or clogged nose, age at introduction of the first complementary food, weaning age, and dietary diversity status; 3) maternal characteristics: body mass index; and 4) household characteristics: wealth index.

#### Ethical considerations

This study was conducted within the framework of the AFRIBIOTA project, which has been approved by the Ethics Committee for Biomedical Research at the Ministry of Public Health in Madagascar (N°104-MSANP/CE - 12/09/2016) and the Institutional Review Board of the Institut Pasteur (2016–06/IRB).

Parents or caregivers were informed about the study and signed the informed consent form before the inclusion of their children. The biological analyses were performed free of charge. Treatments were given to infected and anaemic children according to the national recommendation; the cost of the treatment was covered by the project.

## Results

A total of 490 children between 24 and 59 months of age were included in the AFRIBIOTA project; 450 of these children were recruited in the community setting and were eligible for this secondary analysis. Of the 450 eligible children, 25 had errors in anthropometric measurements (discrepancies in the classification of nutritional status between field measurements and those calculated by the software), and 11 did not have data on haemoglobin levels; these children were thus excluded from the data analysis (Fig. 1).

Of the 414 children included in this secondary analysis, 45.7% were male, and the median age was 43.9 months (interquartile range IQR 33.3 to 52.3 months). The main characteristics of the study participants are summarized in Table 1.

**Table 1:** Characteristics of the study participants (*n* = 414)

	Occurrence of anaemia			
	Yes (*N* = 101)	No (*N* = 313)	Total (*N* = 414)	*p* value
**Sex**				0.215
Female	49 (21.8%)	176 (78.2%)	225	
Male	52 (27.5%)	137 (72.5%)	189	
**Age (months)**				< 0.001
Median (IQR)	36 [29.5–45.1]	46.4 [36.6–53.2]	43.9 [33.3–52.3]	
**Stunting**				0.006
Yes	58 (31.0%)	129 (69.0%)	187	
No	43 (19.0%)	184 (81.0%)	227	
**History of acute malnutrition**				0.069
Yes	5 (50.0%)	5 (50.0%)	10	
No	96 (23.7%)	308 (76.3%)	404	
**Clogged nose**				0.595
Yes	56 (25.7%)	162 (74.3%)	218	
No	45 (23.0%)	151 (77.0%)	196	
**Runny nose**				1
Yes	64 (24.4%)	198 (75.6%)	262	
No	37 (24.3%)	115 (75.7%)	152	
**Dental caries**				0.097
Yes	31 (19.6%)	127 (80.4%)	158	
No	70 (27.3%)	186 (72.7%)	256	
**Dermatitis**				0.940
Yes	7 (27.0%)	19 (73.0%)	26	
No	94 (24.2%)	294 (75.8%)		
**Cough**				1
Yes	37 (24.5%)	114 (75.5%)	151	
No	64 (24.3%)	199 (75.8%)	263	
**Dietary diversity score**				1
Low	37 (24.7%)	113 (75.3%)	150	
Adequate	64 (19.5%)	200 (80.5%)	264	
**Weaning age**				0.856
≤ 12 mo	8 (19.5%)	33 (80.5%)	41	
12–24 mo	19 (23.2%)	63 (76.8%)	82	
≥ 24 mo	60 (23.4%)	196 (76.6%)	256	
Missing data	14	21	35	
**Age at the introduction of first food**				0.733
< 6 mo	32 (23.0%)	107 (77.0%)	139	
≥ 6 mo	68 (25.1%)	203 (74.9%)	271	
Missing data	1	3	4	
**Mother’s nutritional status**				0.740
Not underweight	85 (32.5%)	258 (67.5%)	261	
Underweight	14 (24.5%)	43 (75.5%)	75	
Missing data	2	12	14	
**Intestinal carriage of at least one parasite**				0.795
Yes	85 (23.5%)	274 (76.5%)	359	
No	13 (26.5%)	36 (73.5%)	49	
**Presence of** ***Entamoeba histolytica***				0.080
No	68 (21.4%)	249 (78.5%)	317	
Yes	20 (32.8%)	41 (67.2%)	61	
Missing data	13	23	36	
**Faecal anti-trypsin**				0.960
Normal	72 (24.0%)	227 (76.0%)	299	
Elevated	17 (23.0%)	57 (77.0%)	74	
Missing data	12	29	41	
**Faecal calprotectin**				0.001
Normal	43 (23.7%)	196 (76.3%)	239	
Too high	45 (33.8%)	88 (66.2%)	133	
Missing data	13	29	42	
**Blood level of citrulline**				0.284
Too low	1 (20.0%)	4 (80.0%)	5	
Normal	96 (25.0%)	288 (75.0%)	384	
Too high	0 (0.0%)	8 (100%)	8	
Missing data	4	13	17	
**Iron deficiency**				< 0.001
Yes	52 (58.4%)	37 (41.6%)	89	
No	48 (14.9%)	275 (85.1%)	323	
Missing data	1	1	2	
**Household socioeconomic level**				0.506
Low	72 (26.2%)	203 (73.8%)	275	
Middle	26 (21.0%)	98 (79.0%)	124	
High	3 (20.0%)	12 (80.0%)	15	

Thirty-four percent of the included children (34%) had an age of introduction of the first food before the sixth month, 10.8% had a weaning age of 12 months or less, and 21.6% were weaned between 12 and 24 months of age. Our data showed that 2.4% of the children had had a previous episode of malnutrition and that 45.2% were currently stunted. The proportion of children with low dietary diversity scores was 36.2%. At the time of inclusion, 63.3% of the children had a runny nose, 38.2% had dental caries, and 36.5% had a cough. Fourteen percent (14%) of the children’s mothers were considered underweight (BMI < 18.5 kg/m^2^). Approximately 66.4% of the children came from households with low socioeconomic scores.

Among the participants who provided stool samples (*n* = 408/414), the proportion of children infected with at least one of the investigated parasites was 88%. The most commonly identified parasites were *Trichuris trichura* (67.4%) and *Ascaris lumbricoides* (53.7%).

Thirty-six percent (36%) of the children had elevated stool calprotectin levels, 96.7% had normal citrulline values, and 21.6% showed iron deficiency.

The proportion of children with anaemia was 24.4, and 9.4% of the participants had iron deficiency-related anaemia.

The results of the logistic regression analysis are presented in Table 2. They show that child age, faecal calprotectin level and iron status are independently associated with the occurrence of anaemia. We found that older children were less likely to have anaemia than younger children (OR_a_: 0.95; 95% CI: 0.93–0.98). Children with high levels of faecal calprotectin and iron deficiency were more likely to show anaemia than those with normal faecal calprotectin levels and those with no iron deficiency; the adjusted odds ratios were 2.5 (1.42–4.41) and 6.14 (3.39–11.13), respectively. We found an interaction between age and iron deficiency, the association between iron deficiency and the occurrence of anaemia differs according to age. Irrespective of age, the presence of iron deficiency is associated with a high risk of anaemia, with a higher risk in the older age groups (for children < 43.8 months, the OR was 6.07 [2.58–14.26], for those ≥43.8 months, the OR was 8.7 [4.0–19.0]).

**Table 2:** Logistic regression analysis between the characteristics of children and the occurrence of anaemia among children 24–59 months of age

Variable	Anaemia		Crude OR	Adjusted OR	***p*** value
	Yes (*N* = 81)	No (*N* = 268)			
**Age**^a^ **(in months)**	37.2 [29.7–45.1]	46.5 [36.3–53.8]	0.94 (0.92–0.96)	0.95 (0.93–0.98)	< 0.001
**Faecal calprotectin level**					
Normal	38 (16.8%)	187 (83.2%)	1	1	
Too high	43 (34.7%)	81 (65.3%)	2.33 (1.43–3.8)	2.5 (1.42–4.41)	0.002
**Iron deficiency**					
No	40 (14.5%)	236 (85.5%)	1	1	
Yes	41 (56.1%)	32 (43.9%)	8.05 (4.78–13.56)	6.14 (3.39–11.13)	< 0.001
**Presence of** ***Entamoeba histolytica***					
No	63 (21.5%)	229 (78.5%)	1		
Yes	18 (31.6%)	39 (68.4%)	1.79 (0.98–3.25)		
**Dental caries**					
Yes	52 (24.7%)	158 (75.2%)	0.65 (0.4–1.05)		
No	29 (20.9%)	110 (79.1%)	1		
**History of acute malnutrition**					
Yes	5 (50.0%)	5 (50.0%)	3.21 (0.91–11.32)		
No	76 (22.4)	263 (77.6%)	1		
**Stunting**					
Yes	44 (29.1%)	107 (70.9%)	1.92 (1.22–3.03)		
No	37 (18.7%)	161 (81.3%)	1		
**Age**^a^**Iron deficiency**				1.07 (1.01–1.013)	0.026

^a^: median and interquartile range; 95% CI: confidence interval at 95%. The variables integrated into the backwards stepwise logistic regression were age, faecal calprotectin level, iron status, presence of stunting or not, presence or absence of Entamoeba spp., history of acute malnutrition and presence or absence of dental caries

## Discussion

Our study conducted in children 24–59 months of age living in poor neighbourhoods of the city of Antananarivo aimed to assess factors associated with anaemia. Approximately one quarter (24.4%) of the children were anaemic. Older age was a protective factor, whereas iron deficiency and gut inflammation (high faecal calprotectin levels) were risk factors for anaemia.

Consistent with previous data, our results suggest that older children were less likely to be anaemic than younger children [24–26]. A higher prevalence of anaemia in younger children could be caused by failure to meet the particularly high demand for iron during this period of rapid growth; this might result in nutritional gaps that increase the risk of iron deficiency and anaemia [11].

We found that children with high levels of faecal calprotectin were more likely to suffer from anaemia. Faecal calprotectin is a biomarker of gut inflammation [27]. Calprotectin is a cytoplasmic calcium-binding protein that is found in neutrophils, monocytes and early-stage macrophages. Measurement of calprotectin levels in stool is currently used to diagnose inflammatory bowel diseases, but it has also been used to evaluate the possible presence of other disease states that present with an inflammatory component [28], such as *Schistosoma mansoni* infection [29] and colorectal inflammation [30]. As in our study, an association between anaemia and inflammation was found in preschool children who participated in the BRINDA study [25]; that study reported that inflammation was associated with anaemia in the groups with high and very high infection burdens but not in the groups with low or moderate infection burdens. These findings are consistent with our results, as our study was conducted in disadvantaged neighbourhoods in which many children had a high infection burden, illustrated by the fact that almost all of the children included in our study (88%) were infected by at least one intestinal parasite. However, we failed to find an association between the presence of intestinal parasites and the occurrence of anaemia. Despite the fact that intestinal parasitic carriage was not significantly associated with anaemia, we detected at least one intestinal parasite in 86.7% of anaemic patients. Therefore, efforts to protect children living in these underprivileged neighbourhoods from infection by these parasites are urgently needed.

In our study, iron deficiency increased the risk of developing anaemia. This is consistent with the fact that iron is needed for erythropoiesis and that failure to meet the body’s demand for iron can lead to iron-restricted erythropoiesis. Inadequate iron supply can result from either nutritional iron deficiency or iron restriction during infection and inflammation [31]. Iron deficiency has long been assumed to contribute to approximately 50% of anaemia cases globally [32]; however, a study conducted across a range of countries with varying rankings on the Human Development Index showed that only approximately one quarter of anaemia cases were associated with iron deficiency, while the rest had other aetiologies. We found that in underprivileged neighbourhoods of Antananarivo, iron deficiency and gut inflammation were associated with the occurrence of anaemia. These results suggest that local inflammation may cause gastrointestinal malabsorption of iron [33] and subsequently lead to anaemia.

We found a proportion of 24.4% of children with anaemia, a value that is 50% lower than the national prevalence of the disease. This difference might be explained by the particular characteristics of the study population, as we assessed a group of children who lived in an underprivileged area and were specifically selected according to their nutritional status. There might thus be a bias in our study compared to the estimate of anaemia prevalence nationwide, which was determined using a representative sample. Our study was conducted in an underprivileged area in which many nutritional interventions (distribution of meals and flour, sensitization, and other interventions) are conducted, and this could have led to an improvement in the children’s diets that influenced their Hb levels.

Our findings have implications for strengthening the existing public health and nutrition efforts in Madagascar intended to benefit children living in underprivileged urban areas. Under the third nutrition plan for 2017 to 2021 (*Plan National d’Action pour la Nutrition PNAN III 2017–2021)* [34], numerous interventions to improve maternal and child nutrition have been planned; they include activities that will reduce the prevalence of anaemia and micronutrient deficiency (mainly iron) in children under five, such as iron supplementation, deworming, malaria prevention during pregnancy and promotion of home food fortification (provision of multiple micronutrient powders (MNPs)). According to our results, the current strategies should be combined with the prevention and treatment of infectious diseases that might lead to inflammation, such as parasitic infections, which are very common in our population study. This could be accomplished by promotion of WASH activities, deworming, and other interventions. These combined strategies will address all the factors associated with anaemia and will optimize iron intervention efforts, as iron deficiency is known to be multifactorial.

Our study has several limitations. The study population is not representative of the entire population of Antananarivo, the capital city; however, the results do illustrate the anaemia situation in underprivileged areas of Antananarivo, which represents approximately 20% of the total population of the city. Some data, such as disease history and history of acute malnutrition, might have introduced recall bias; thus, we limited our survey of disease history to inquiring about serious illnesses that required hospitalization (for diarrhoea or respiratory disease) in the year prior to the study. Nevertheless, the findings of this study will enable public health decision-makers to improve their policy actions to fight anaemia. Such policy actions should be focused on decreasing the burden of infectious diseases and on improving young children’s dietary quality and micronutrient intake.

## Supplementary Information

**Additional file 1.**

## Data Availability

All data generated or analysed during this study are included in the published article and its supplementary information files.

## Abbreviations

AAT: Alpha Antitrypsin
AFRIBIOTA: The pathophysiology of environmental enteric dysfunction in Madagascar and the Central African Republic project
BMI: Body Mass Index
CHUJRA: Centre Hospitalo-Universitaire Joseph Ravoahangy Andrianavalona
CMIA: Chemiluminescent Microparticle Immunoassay
CSMI: Centre de Santé Maternelle et Infantile de Tsaralalana
CRP: C-reactive protein
DDS: Dietary Diversity Score
EED: Environmental Enteric Dysfunction
EDTA: Ethylenediamine Tetraacetic Acid
Hb: Haemoglobin
HIV: Human Immunodeficiency Virus
INSTAT: Institut National de la Statistique
IPM: Institut Pasteur de Madagascar
IQR: Interquartile range
MMP: Multiple Micronutrient Powders
OMS: Organisation Mondiale de la Santé
ONN: Office National de la Nutrition
OR: Odds ratio
PCA: Principal Component Analysis
PNAN: Plan National d’Action pour la Nutrition
PreSAC: Preschool-aged children
SD: Standard deviation
UNESCO: United Nations Educational, Scientific and Cultural Organization
UNICEF: United Nations Children’s Fund
USA: United States of America
USAID: United States Agency for International Development
USD: United States dollar
WHO: World Health Organization
